# Neuroprotective Potential of *Stevia rebaudiana* and *Stachys sieboldii*: Effects on Oxidative Stress and Locomotor Activity in Male Rats Fed a High-Fat, High-Sucrose Diet

**DOI:** 10.3390/biology14040359

**Published:** 2025-03-31

**Authors:** Yelena Pozdnyakova, Aigul Murzatayeva

**Affiliations:** Department of Biomedicine, Karaganda Medical University, Karaganda 10008, Kazakhstan; murzatayeva@qmu.kz

**Keywords:** high-sugar diet, HFHS, oxidative stress, lipid peroxidation, *Stevia rebaudiana*, *Stachys sieboldii*

## Abstract

A high-fat, high-sugar diet (HFHS) is known to exacerbate oxidative stress and behavioral dysfunctions, increasing susceptibility to cognitive decline. This study aimed to evaluate the effects of *Stevia rebaudiana* and *Stachys sieboldii* on lipid peroxidation and behavioral alterations in rats fed an HFHS diet. Forty Wistar rats were divided into four groups: a control group on a standard diet, an HFHS group, and two experimental groups receiving HFHS supplemented with either Stevia or Stachys for 30 days. Behavioral responses were assessed using the Open Field Test, and oxidative stress markers (malondialdehyde, conjugated dienes, ketodienes, and Schiff bases) were measured in brain homogenates. Stevia significantly reduced oxidative stress markers by 30–51%, whereas Stachys decreased hyperactive locomotor behavior by 63–68%. Correlation analysis revealed strong associations between lipid peroxidation and behavioral parameters. These findings suggest that Stevia is more effective in reducing oxidative stress, while Stachys better regulates excessive locomotion. Both plant-based supplements exhibit neuroprotective potential, with distinct mechanisms of action, supporting their use as dietary interventions to mitigate the effects of an HFHS diet on brain function.

## Simple Summary

A high-fat, high-sugar (HFHS) diet is associated with elevated oxidative stress and behavioral changes, increasing the risk of neurodegenerative disorders. This study investigated whether *Stevia rebaudiana* and *Stachys sieboldii* supplementation could mitigate these effects in adult Wistar rats over 30 days. HFHS feeding significantly heightened lipid peroxidation markers (malondialdehyde, conjugated dienes, ketodienes, and Schiff bases; all *p* < 0.0001) and increased locomotor activity by 107–126%. Stevia supplementation reduced oxidative stress indicators by 30–51% (*p* < 0.0001), whereas Stachys more prominently decreased hyperactive behavior, lowering vertical and horizontal activity by 63–68% (*p* < 0.0001). Grooming and defecation measures remained unchanged. These results suggest that Stevia is more effective at alleviating oxidative damage, while Stachys primarily modulates excessive locomotion. By selectively targeting distinct facets of HFHS-induced dysfunction, both plant-based supplements demonstrate potential as natural interventions to support brain health under metabolically stressful dietary conditions.

## 1. Introduction

Diet and nutrition play a crucial role in maintaining the health and quality of life of older adults. With aging, metabolic processes undergo significant changes, necessitating specific dietary adjustments [1]. The key principles of effective nutrition for the elderly include caloric reduction due to decreased physical activity and a slower energy metabolism, increased protein intake to support muscle mass, as well as higher consumption of vitamins and minerals to maintain bone health and immune function. Additionally, a higher intake of dietary fiber is recommended to promote digestive system function [2].

Moreover, older adults often experience physiological changes such as reduced appetite, alterations in taste perception, and difficulties with chewing and swallowing, all of which influence their dietary habits and overall health status [3]. The presence of chronic diseases, including diabetes, cardiovascular diseases, and osteoporosis, further necessitates dietary modifications, requiring the restriction or elimination of certain food products [4,5].

In the United States, elderly individuals face challenges in maintaining dietary diversity. The typical American diet is characterized by high consumption of low-cost processed foods, fast food, sugar, and saturated fats [6]. This dietary pattern contributes to an increased prevalence of obesity, diabetes, and cardiovascular diseases among the elderly population. In recent years, there has been a shift towards healthier eating habits, with a greater emphasis on fresh produce, fish, and nuts. However, the widespread consumption of highly processed foods remains a significant public health concern [7].

In African countries such as Ethiopia, older adults often face nutritional challenges due to economic constraints and climatic conditions [8]. Their diet primarily consists of cereals, including teff, maize, and sorghum, as well as legumes such as lentils and beans. Meat and fish are consumed in limited quantities, mainly due to their high cost. The lack of diverse and nutrient-rich foods in their diet leads to deficiencies in essential vitamins and minerals, exacerbating health problems among the elderly population [9].

In Russia, the dietary habits of older adults differ significantly from those of younger generations. Their diet predominantly includes bread, pasta, and dairy products. Meat consumption is lower compared to younger individuals, while traditional dishes such as soups and porridges are commonly consumed. These foods are rich in carbohydrates but may lack sufficient vitamins and minerals. Additionally, there is a noticeable decline in the intake of fruits and vegetables, which negatively affects overall health status [10].

In Kazakhstan, the diet of older adults includes a significant amount of meat, dairy products, bread, and flour-based foods. Vegetables and fruits are also present in their diet, though their consumption is considerably lower than in European countries. In rural areas, traditional dishes such as baursak, beshbarmak, and kumis remain an essential part of the local diet [11].

The dietary patterns of older adults, particularly those dominated by fats and carbohydrates, have a significant impact on their health and cognitive function. With aging, the human body undergoes numerous changes affecting both metabolism and the function of internal organs [12].

Fats and carbohydrates serve as primary energy sources for the body. However, excessive or imbalanced consumption of these macronutrients can lead to various adverse health effects [13]. Notably, high intake of fats—especially saturated and trans fats—is strongly associated with obesity, atherosclerosis, type 2 diabetes, and cardiovascular diseases [14,15]. These conditions, in turn, can severely deteriorate overall health and reduce life expectancy in older individuals.

The detrimental effects of a high-calorie diet rich in fats and simple carbohydrates on cognitive function remain a pressing issue, prompting the development of various intervention strategies. One promising approach involves the use of bioactive supplements containing antioxidants, polyphenols, and other neuroprotective compounds that help mitigate oxidative stress and inflammation in the brain [16].

Beyond detecting the overall increase in reactive oxygen species under metabolic stress induced by a high-fat, high-sucrose diet, an important approach involves analyzing specific stages of lipid peroxidation in the brain. To achieve this, multiple biomarkers are traditionally assessed, allowing a comprehensive evaluation of the entire cascade of membrane damage. Thus, the formation of conjugated dienes and ketodienes reflects the initial and intermediate stages of polyunsaturated fatty acid oxidation, when free radicals have already triggered chain reactions in phospholipids [17].

The increase in malondialdehyde (MDA) represents a later stage: this stable degradation product of peroxidized radicals is capable of forming cross-links with proteins and nucleic acids, thereby exacerbating cellular dysfunction [18]. Schiff bases represent the “final” compounds that arise from the interaction of aldehyde groups of oxidized lipids with the amino groups of proteins or amino acids and serve as markers of the most severe oxidative damage [19].

This comprehensive approach to identifying different phases of oxidative stress allows the tracking of its mechanism—from the initiation of membrane structure disruption to the formation of toxic aldehyde metabolites. When evaluating the neuroprotective potential of natural agents, it is essential to understand not only the “final” effects but also the intermediate pathways through which the damaging process unfolds. For this reason, investigating various stages of lipid peroxidation allows a deeper understanding of possible mechanisms for correcting stress-induced changes in the central nervous system [20].

In the search for natural compounds capable of counteracting the adverse effects of high-calorie diets on cognitive function, particular attention has been given to plants with pronounced antioxidant and neuroprotective properties. Among them, the Stachys L. genus (family Lamiaceae) stands out, comprising over 300 species distributed across temperate and tropical regions of the Mediterranean, Asia, the Americas, and southern Africa [21]. Extracts from various Stachys species contain phenolic compounds such as chlorogenic acid, catechin, and rosmarinic acid, which exhibit potent antioxidant activity. Due to these properties, plants of the Stachys genus are widely utilized in the food and pharmaceutical industries [22]. Phytochemical analysis of Stachys tubers has revealed a high content of amino acids, carbohydrates, and glycosylated triterpenoids, whereas alkaloids and flavonoids are present in smaller amounts. Organic acids have been detected in trace concentrations, while tannins, coumarins, and anthraquinones are absent [23]. The significance of phenolic compounds in Stachys has been confirmed by multiple studies demonstrating their therapeutic potential in various neurological disorders. Specifically, compounds such as chlorogenic, syringic, vanillic, and caffeic acids, as well as rutin, catechin, and kaempferol, have been shown to possess anxiolytic and antidepressant properties. These findings highlight the potential of *Stachys sieboldii* tuber powder as a promising agent for mitigating cognitive and emotional disturbances [24,25].

Moreover, recent evidence indicates that Stachys species can confer direct neuroprotective benefits by reducing oxidative stress and modulating behavior in rodent models of neurological dysfunction [26]. In a seizure-based model, *Stachys lavandulifolia* exhibits anxiolytic and antidepressant effects, while tubers of *Stachys sieboldii* protect against ischemia-induced memory impairments via antioxidant mechanisms [27].

Another plant with pronounced bioactive properties is *Stevia rebaudiana* Bert. (family Asteraceae). This perennial shrub is widely cultivated in South America, Asia, and other regions worldwide and is primarily used as a natural sweetener [28]. The key bioactive compounds of *Stevia rebaudiana* are diterpene glycosides, including stevioside, rebaudiosides A, B, C, D, E, and F, steviolbioside, and dulcoside A, which impart an intense sweetness that exceeds that of sucrose by several hundred times [29]. Beyond its sugar-substituting properties, *Stevia rebaudiana* is rich in proteins, amino acids, lipids, vitamins, and minerals. Phytochemical analysis has revealed a significant presence of tannins, alkaloids, glycosides, saponins, sterols, and triterpenes, which contribute to its broad spectrum of biological activities. Extracts of *Stevia rebaudiana* have demonstrated antimicrobial, antihypertensive, anti-inflammatory, antitumor, hepatoprotective, and immunomodulatory effects in both in vitro studies and animal experiments [30]. In addition to these well-established properties, several investigations suggest that Stevia exerts neuroprotective effects by mitigating oxidative damage in the central nervous system and restoring balance in metabolic disturbances linked to diet-induced obesity [31]. In an epileptic rat model, Stevia extract improved oxidative stress markers and reduced seizure severity, while stevioside attenuated lipid peroxidation and nitric oxide production in the brains of overfed zebrafish [32].

The aim of this study is to conduct a comparative analysis of the effectiveness of *Stevia rebaudiana* and *Stachys sieboldii* as bioactive supplements in mitigating the adverse effects of a high-fat, high-carbohydrate diet (HFHS) on the central nervous system, with a particular focus on their potential neuroprotective and antioxidant properties.

## 2. Materials and Methods

### 2.1. Material Preparation

The *Stachys sieboldii* root powder used in this study was produced at the Phytochemistry Holding (Karaganda, Kazakhstan). To ensure the purity of the raw material, the roots were washed three times with running water to remove contaminants such as sand and dust particles. Subsequently, the rhizomes were subjected to lyophilization for 72 h and then ground into a fine powder. The resulting powder was stored at −70 °C until its incorporation into the experimental diets for rodents.

The powdered organic extract of *Stevia rebaudiana* leaves was purchased from an online retailer (SweetLeaf, Gilbert, AZ, USA).

The dosage was calculated based on the U.S. Food and Drug Administration (FDA) recommended acceptable daily intake (5 mg/kg) [33]. These doses were determined for each group by multiplying the acceptable daily intake by the average weight of the rats and then dividing by the mean daily fluid intake per group. Dosages were adjusted weekly to account for changes in body weight and fluid consumption.

### 2.2. Animal Experiments and Diets

Mature male Wistar rats (*n* = 40) aged 18 months, corresponding to approximately 60 years in humans [34], were used in this study. This age was chosen to model age-related changes associated with dietary stress and oxidative processes in the brain. The animals were randomly assigned to four experimental groups (*n* = 10 per group). The control group (Intact) received a standard diet, while the second group (HFHS) was fed a high-fat, high-sucrose (HFHS) diet. In the third group (HFHS + Stachys), *Stachys sieboldii* root powder (5 g/kg) was added to the HFHS diet, and in the fourth group (HFHS + Stevia), *Stevia rebaudiana* leaf powder (5 g/kg) was introduced instead of *Stachys sieboldii*.

The sample size was determined based on preliminary calculations and power analysis, which demonstrated that 10 animals per group were sufficient to detect statistically significant differences between the groups. The power analysis confirmed that the probability of a Type II error was minimal.

All animals included in the study were male Wistar rats weighing 200–350 g, with no visible health abnormalities. Animals with deviations in body weight, signs of illness, or evident physical impairments were excluded from the experiment. No animals were excluded during the study, and all included rats were accounted for in the statistical analysis. Initially, both male and female rats were recruited, as one objective was to examine potential sex differences in response to the high-fat, high-sucrose diet and plant-based supplements. However, subsequent analyses revealed significant differences between males and females in oxidative stress levels and behavioral responses. As a result, it was decided to separate the data by sex and present them in separate articles to avoid confounding factors and ensure more precise interpretation. The present study, therefore, focuses exclusively on the data obtained from male rats.

Animals were randomly assigned to groups using a computerized random number generator, minimizing potential biases related to individual physiological characteristics. A partial blinding method was applied: while group allocation was randomized, the experimenter assessing behavioral parameters was unaware of the dietary interventions. Biochemical analyses were conducted using coded samples, eliminating any subjective bias in data interpretation.

All animals were housed under standard vivarium conditions to control potential confounding factors: temperature was maintained at 18 ± 2 °C, humidity at 55% ± 5%, and a 12 h light/dark cycle was ensured. The rats had free access to food and water throughout the experiment. Before the study began, they underwent a mandatory seven-day quarantine period, during which their general health status, adaptation to housing conditions, and stability of food and water consumption were monitored. This step was crucial for minimizing any stress effects related to environmental changes that could influence the experimental outcomes.

### 2.3. Diet Composition

The experimental diets for rodents were formulated with different ingredient combinations, creating four distinct groups.

- Control group (Intact): Corn—20.0%, rice—20.0%, bone meal—12.0%, sucrose—0.0%, soy oil—7.5%, lard—0.0%, gluten—20.0%, salt—0.35%, mineral mix—3.5%, vitamin mix—1.65%, inert material—15.0%.
- High-fat, high-sucrose diet group (HFHS): The diet composition was modified by reducing the corn content and eliminating soybean oil. Instead, pork lard was incorporated, increasing the lipid content. Additionally, sucrose was added to elevate carbohydrate levels. Corn—8.0%, rice—20.0%, bone meal—12.0%, sucrose—10.0%, soy oil—0.0%, lard—20.0%, gluten—20.0%, salt—0.35%, mineral mix—3.5%, vitamin mix—1.65%, inert material—4.5%
- HFHS + Stachys group: This diet included an additional 5 g/kg of *Stachys sieboldii* root powder, while maintaining the overall macronutrient balance similar to the HFHS group.
- HFHS + Stevia group: In this group, 5 g/kg of *Stevia rebaudiana* leaf powder was added to the diet instead of *Stachys sieboldii*, keeping the macronutrient composition consistent with the HFHS group [25].

The experiment lasted 30 days.

### 2.4. Determination of Physiological Parameters

**Figure 1:**
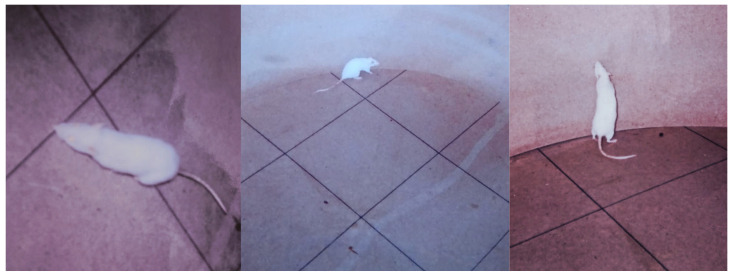
Representative images of rats during the Open Field Test across experimental groups.

To assess changes in behavioral responses, the Open Field Test was conducted on Day 30 of the experiment. The test was performed in a circular arena with a diameter of 150 cm, divided into 16 sectors and enclosed by opaque walls 50 cm high (Figure 1). Each rat was carefully placed in the center of the arena, held by the tail, and observed for 2 min. During this period, the number of crossed sectors (horizontal activity), frequency of vertical movements, and instances of grooming, defecation, and urination were recorded. After the observation period, the rat was removed, and the arena was thoroughly cleaned to eliminate olfactory traces that could influence subsequent animals [34].

### 2.5. Euthanasia and Brain Tissue Collection

All animals were euthanized using CO_2_ asphyxiation followed by cervical dislocation, ensuring a humane and ethically approved method of euthanasia. The whole brain was then extracted from each rat for subsequent biochemical analysis.

### 2.6. Biochemical Analysis in Brain Homogenates

For biochemical assessments, fresh brain tissue was collected and rinsed with chilled physiological saline. A 10% (*w*/*v*) homogenate was prepared by suspending the samples in 0.1 M phosphate buffer (pH 7.4). The homogenates were then centrifuged at 10,000 rpm at 4 °C for 15 min using a refrigerated centrifuge. The supernatant was immediately frozen in liquid nitrogen and stored at −80 °C until further analysis.

### 2.7. Determination of Conjugated Dienes, Ketodienes, and Schiff Bases

A 0.3 mL aliquot of the supernatant was diluted 1:33 with phosphate buffer (pH 7.4). Then, 1 mL of the diluted sample was transferred to a stoppered test tube, and 9 mL of a heptane–isopropanol mixture (1:1, *v*/*v*) was added. The mixture was vigorously shaken for 1 min, followed by centrifugation at 3000 rpm for 5 min. After an additional 1 min phase separation, 3 mL of the upper heptane layer was collected, and optical density measurements were performed. The absorbance at 232 nm was recorded to determine conjugated dienes, while ketodienes were quantified by measuring absorbance at 268 nm. The Schiff base levels were evaluated by calculating the optical density ratio at 400 nm and 220 nm (E_400_/E_220_) [35].

### 2.8. Measurement of Malondialdehyde (MDA)

For the analysis, 0.3 mL of the supernatant was mixed with 2.4 mL of 1/12 N H_2_SO_4_ and 0.3 mL of 10% phosphotungstic acid, followed by thorough mixing. After 10 min of incubation, the samples were centrifuged at 3000 rpm for 10 min, and the resulting precipitate was washed twice with 1 mL of distilled water. The washed precipitate was then dissolved in 3 mL of water, and 1 mL of freshly prepared TBA solution in acetic acid was added. This solution was obtained by dissolving 80 g of TBA in a mixture of 5 mL of water and 5 mL of glacial acetic acid under heating.

To initiate the colorimetric reaction, the test tubes were incubated at 96 °C for 60 min, followed by rapid cooling in cold water. To eliminate possible turbidity before spectrophotometric analysis, the samples were further centrifuged at 3000 rpm for 15 min. The optical density was measured at 532 nm using 10 mm cuvettes, with distilled water used as a reference. The MDA concentration was calculated using the formula C = E/ξ, where E represents the optical density, ξ is the molar extinction coefficient (1.56 × 10^−5^), and C is expressed in micromoles per milliliter of supernatant [36].

The obtained samples were analyzed using a UV–Vis PD-303UV digital spectrophotometer (APEL, Saitama, Japan).

### 2.9. Statistical Analysis

Statistical data processing was performed using GraphPad Prism 8.0.1 software. The Shapiro–Wilk, Kolmogorov–Smirnov, and D’Agostino–Pearson tests were used to assess the normality of data distribution. In cases where the data did not follow a normal distribution, both parametric and nonparametric methods were applied.

For group comparisons, the following statistical methods were used: one-way analysis of variance (ANOVA) with Tukey’s post hoc test—applied for parametric data that followed a normal distribution. Kruskal–Wallis test with Dunn’s post hoc test—used for nonparametric data, allowing the detection of significant differences between groups when data deviated from a normal distribution.

Pearson’s correlation coefficient (r) was used to assess the strength and direction of associations between behavioral parameters and biochemical markers. The correlation analysis was performed separately for the groups receiving *Stachys sieboldii* and *Stevia rebaudiana*, enabling a comparison of their distinct mechanisms of action.

In all cases, a statistical significance level of *p* < 0.05 was considered. The use of both parametric and nonparametric approaches ensured the accuracy of data interpretation, allowing the identification of significant differences between groups and the determination of relationships between the studied parameters.

## 3. Results

### 3.1. Changes in Behavioral Responses in Rats Under Dietary Exposure and Correction with Stachys and Stevia

**Figure 2:**
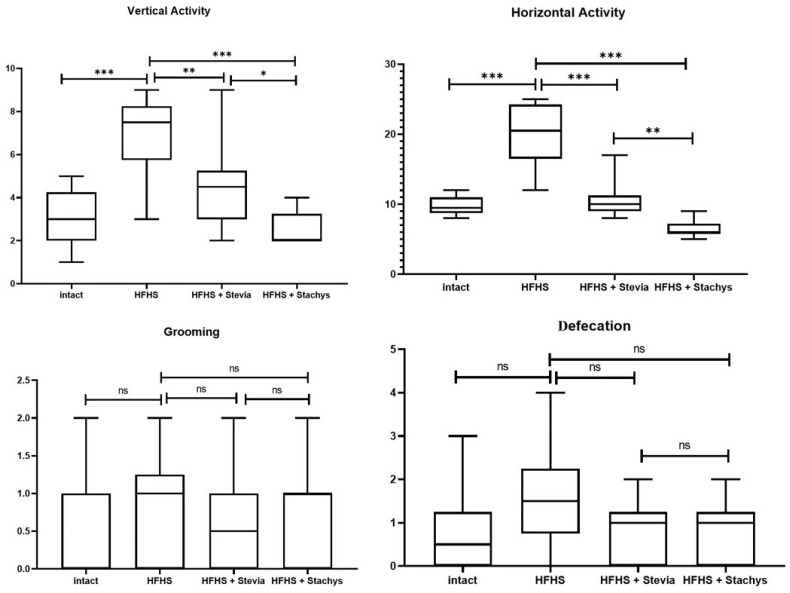
Comparison of behavioral parameters—vertical activity, horizontal activity, grooming, and defecation—across four groups: rats on a standard diet (Intact), on a high-fat, high-sucrose (HFHS) diet, and on HFHS supplemented with either *Stevia rebaudiana* or *Stachys sieboldii* (for group comparisons of vertical and horizontal activity, one-way ANOVA with Tukey’s post hoc test was used; for group comparisons of grooming and defecation, the Kruskal–Wallis test with Dunn’s post hoc test was applied). Symbols: * *p* < 0.05, ** *p* < 0.01, *** *p* < 0.001, ns: not significant.

The results of statistical analysis of vertical activity in adult rats indicate that in the group of animals receiving a high-fat, high-sucrose diet (HFHS), this parameter increased by approximately 107% (*p* < 0.0001) compared to the control group (Intact). The addition of Stevia to this diet (HFHS + Stevia) led to a reduction in vertical activity by approximately 47% (*p* < 0.0001) compared to the HFHS group. The inclusion of Stachys in the diet (HFHS + Stachys) resulted in an even more pronounced decrease in this parameter—by approximately 68% (*p* < 0.0001)—relative to the HFHS group (Figure 2).

Analysis of horizontal activity revealed that in rats receiving the HFHS diet, this parameter was approximately 126% higher (*p* < 0.0001) compared to the control group (Intact). The addition of Stevia (HFHS + Stevia) resulted in a reduction in horizontal activity by approximately 34% (*p* = 0.0078) relative to the HFHS group. The introduction of Stachys (HFHS + Stachys) led to an even more substantial decrease of approximately 63% (*p* < 0.0001) compared to the HFHS group. Statistical analysis of grooming behavior showed that in the HFHS-fed group, this parameter did not differ significantly from the control level (Intact) (*p* > 0.05). Similarly, the inclusion of Stevia (HFHS + Stevia) and Stachys (HFHS + Stachys) did not result in statistically significant changes in grooming behavior compared to the HFHS group (*p* > 0.05). A similar trend was observed in the assessment of defecation: in the HFHS group, its level did not significantly differ from that of the control animals (Intact) (*p* > 0.05). The addition of Stevia (HFHS + Stevia) or Stachys (HFHS + Stachys) also did not have a statistically significant effect on this parameter compared to the HFHS group (*p* > 0.05) (Figure 2).

### 3.2. Levels of Lipid Peroxidation Products in Rat Brain Homogenates

The statistical analysis of conjugated diene concentrations in the brain of adult rats demonstrated that in the group receiving a high-fat, high-sucrose diet (HFHS), the levels of conjugated dienes increased by approximately 101% (*p* < 0.0001) compared to the control group (Intact). The addition of Stevia to this diet (HFHS + Stevia) resulted in a reduction in this parameter by approximately 43% (*p* < 0.0001) compared to the HFHS group. Similarly, the inclusion of Stachys (HFHS + Stachys) in the diet led to a decrease in conjugated diene levels by approximately 28% (*p* < 0.0001) relative to the HFHS group.

**Figure 3:**
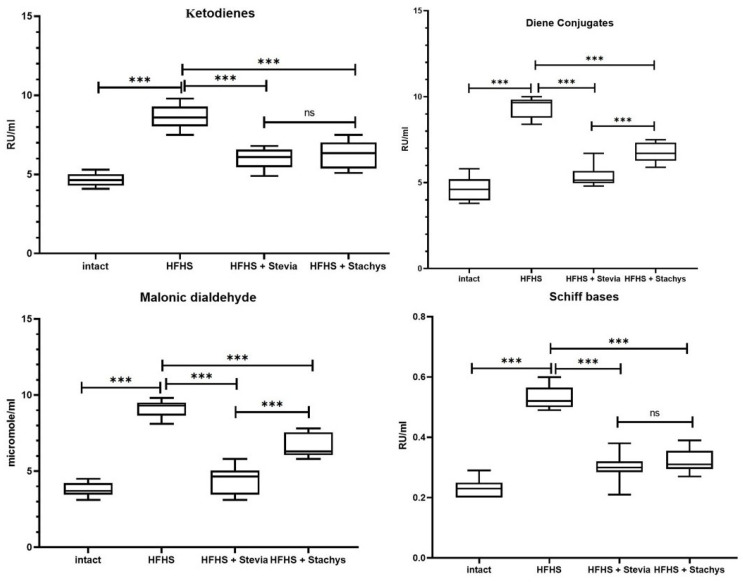
Quantitative assessment of lipid peroxidation markers (malondialdehyde, conjugated dienes, ketodienes, and Schiff bases) in brain homogenates from rats receiving a standard diet, a HFHS diet, and HFHS diets supplemented with *Stevia rebaudiana* or *Stachys sieboldii* (for group comparisons, one-way ANOVA with Tukey’s post hoc test was used). Symbols: *** *p* < 0.001, ns: not significant.

Regarding ketodiene concentrations, rats fed the HFHS diet exhibited an increase of approximately 85% (*p* < 0.0001) compared to the control group (Intact). The supplementation with Stevia (HFHS + Stevia) led to a reduction in ketodiene levels by approximately 30% (*p* < 0.0001) relative to the HFHS group. The introduction of Stachys (HFHS + Stachys) also resulted in a decrease in ketodiene concentrations by approximately 27% (*p* < 0.0001) compared to the HFHS group (Figure 3).

The analysis of malondialdehyde (MDA) concentrations revealed that in the HFHS group, MDA levels exceeded control values by approximately 142% (*p* < 0.0001). The inclusion of Stevia in the diet (HFHS + Stevia) contributed to a reduction in this parameter by approximately 51% (*p* < 0.0001) compared to the HFHS group without supplementation. The administration of Stachys (HFHS + Stachys) also led to a decrease in MDA levels by approximately 27% (*p* < 0.0001) relative to the HFHS group. An evaluation of Schiff bases demonstrated that their concentration in the HFHS group increased by approximately 130% (*p* < 0.0001) compared to the control group (Intact). In the HFHS + Stevia group, a reduction in Schiff base levels by approximately 43% (*p* < 0.0001) was observed compared to the HFHS group. In animals receiving Stachys in addition to the high-fat, high-sucrose diet (HFHS + Stachys), Schiff base levels decreased by approximately 39% (*p* < 0.0001) relative to the HFHS group (Figure 3).

### 3.3. Correlation Analysis of Behavioral and Biochemical Parameters in Experimental Group

Pearson’s correlation analysis revealed differences in the relationships between behavioral parameters (vertical and horizontal activity, grooming, and defecation) and biochemical markers (malondialdehyde (MDA) levels and antioxidant indices DK, KD, and SHO) in the groups receiving *Stachys sieboldii* and *Stevia rebaudiana*.

In the group of rats treated with *Stachys sieboldii*, moderate to strong positive correlations were observed among locomotor parameters. Vertical activity exhibited a strong correlation with horizontal activity (r = 0.925) and grooming (r = 0.988), indicating an overall increase in mobility. Defecation also showed a positive correlation with horizontal activity (r = 0.968), which may suggest an impact of increased activity on autonomic responses (Table 1).

The biochemical parameter MDA, an indicator of oxidative stress, exhibited a moderate positive correlation with defecation (r = 0.987), suggesting that increased oxidative stress may be associated with heightened anxiety-related responses. However, the correlation between MDA and grooming was less pronounced (r = 0.794), indicating a weaker direct relationship between oxidative stress and self-regulatory mechanisms in this group.

The correlation analysis of biochemical markers revealed a strong association between the lipid peroxidation marker DK and MDA levels (r = 0.994), suggesting a compensatory activation of antioxidant mechanisms in response to oxidative stress (Table 1).

In the group of rats receiving *Stevia rebaudiana*, a more pronounced synchronization of motor responses was observed. Vertical activity showed an almost perfect correlation with grooming (r = 0.998) and a strong correlation with horizontal activity (r = 0.954), indicating a high level of coordination across different types of movement. Defecation also exhibited strong correlations with vertical activity (r = 0.996) and horizontal activity (r = 0.970), which may suggest a more pronounced activation of stress responses (Table 2).

Unlike the Stachys group, the level of MDA in the Stevia group showed a strong correlation not only with defecation (r = 0.993) but also with grooming (r = 0.977). This may indicate that in the Stevia group, an elevated level of oxidative stress contributes not only to increased anxiety but also to the activation of self-regulation mechanisms.

Biochemical parameters exhibited exceptionally high correlations among themselves (r > 0.99 for DK, KD, and SHO), suggesting a more robust regulation of the antioxidant system in this group.

## 4. Discussion

Various experimental studies confirm that chronic consumption of a high-fat, high-sucrose (HFHS) diet disrupts metabolism and exacerbates oxidative stress in the body. For example, a study by Kobi JBBS et al. (2023) demonstrated that prolonged consumption of fatty and sucrose-rich foods provokes obesity and associated metabolic disturbances, although it is not always accompanied by pronounced inflammation in serum and adipose tissue [37]. Meanwhile, other studies report a more noticeable accumulation of oxidative stress products and enhanced inflammatory responses, particularly when fats and sucrose are combined [38,39]. Specifically, Santiago Santana et al. (2021) indicated that the combined impact of a diet high in saturated fats and psychogenic stress leads to increased neuroinflammation and oxidative damage in the brain, which may modify behavioral responses in rodents [40]. Similarly, Mabrok et al. (2024) observed that obesity induced by a high-calorie diet is accompanied by cognitive decline through oxidative stress mechanisms and metabolic shifts, although translating such behavioral data from rodents to humans requires caution [41].

Our study aligns with these findings, confirming that a high-fat, high-sucrose diet can trigger lipid peroxidation mechanisms and contribute to behavioral changes. At the same time, supplementation with *Stevia rebaudiana* and *Stachys sieboldii* demonstrated the potential to reduce markers of lipid peroxidation in the brain.

Our results indicate that including *Stachys sieboldii* and *Stevia rebaudiana* in the diets of HFHS-fed rats contributes to the reduction in oxidative stress markers and modifies several behavioral responses impaired under hypercaloric conditions. These findings suggest potential neuroprotective and antioxidant properties of these plant-based supplements, which may mitigate the adverse effects of excessive fat and carbohydrate intake on the central nervous system.

Our data are consistent with the study by Azizi et al. (2023), which demonstrated that an extract of *Stachys lavandulifolia* exhibits anxiolytic and antidepressant effects in a rat model of acute seizures, improving behavioral parameters and reducing oxidative stress levels [26]. Similarly, Harada et al. (2015) reported that an extract of *Stachys sieboldii* tubers protects against learning and memory impairments associated with cerebral ischemia through antioxidant mechanisms [27]. These findings suggest that the behavioral stabilization observed in our study may be related to the modulation of oxidative stress pathways by bioactive compounds in *Stachys sieboldii*, which could influence neuronal excitability and synaptic plasticity.

In our study, including Stachys in the diet of HFHS-fed rats led to a reduction in vertical activity by approximately 68% and horizontal activity by 63% compared to the HFHS group, bringing these parameters closer to those observed in the control group. Meanwhile, grooming and defecation parameters did not significantly differ from the HFHS group, indicating a selective effect of Stachys on locomotor activity under hypercaloric dietary conditions. These changes may be explained by the reduction in oxidative stress markers, which influence dopaminergic and serotonergic neurotransmission, leading to alterations in motor activity and emotional status.

Furthermore, we found that lipid peroxidation products such as malondialdehyde (MDA), conjugated dienes, and ketodienes were significantly elevated in the HFHS group, indicating increased oxidative stress in the brain. The addition of Stachys to the diet reduced these parameters compared to the HFHS group: MDA levels decreased by approximately 27%, conjugated dienes by 28%, ketodienes by 27%, and Schiff bases by 39%, although these values remained higher than those in the control group. These findings confirm the antioxidant properties of Stachys, presumably linked to its ability to modulate redox balance, enhance the activity of antioxidant enzymes (superoxide dismutase, glutathione peroxidase), and reduce lipid peroxidation.

Our results complement the findings of Kim et al. (2024), which demonstrated that polyphenolic compounds in *Stachys affinis* extract exhibit significant antioxidant and anti-inflammatory properties, as confirmed by both in vitro experiments and molecular docking analysis [42]. Similarly, Lachowicz-Wiśniewska et al. (2022) reported that extracts from the leaves and flowers of *Stachys palustris* contain high concentrations of polyphenols and exhibit strong antioxidant, antiproliferative, and antidiabetic activity [43]. These findings support the hypothesis that phenolic compounds in Stachys may exert neuroprotective effects by inhibiting oxidative damage and inflammation.

Similar neuroprotective and antioxidant properties have also been identified in *Stevia rebaudiana*, which effectively reduces lipid peroxidation levels in nervous tissue. Our findings align with those of El Nashar et al. (2022), who demonstrated that Stevia extract improved oxidative stress markers in the hippocampus and increased antioxidant enzyme levels in rats with an epilepsy model, reducing both seizure frequency and severity. This supports the neuroprotective properties of Stevia and its ability to mitigate lipid peroxidation in the brain [31].

Similarly, Dandin et al. (2023) reported that stevioside decreases elevated lipid peroxidation and nitric oxide levels in the brains of overfed *Danio rerio*, while simultaneously enhancing antioxidant enzyme activity. These findings suggest that Stevia may help alleviate oxidative stress associated with excessive caloric intake, which is consistent with our observations in HFHS-fed rats [32].

In our study, the levels of lipid peroxidation products, including malondialdehyde (MDA), conjugated dienes, and ketodienes, were elevated in the HFHS group, indicating increased oxidative stress within the brain. The addition of *Stevia rebaudiana* to the diet resulted in a reduction in these markers compared to the HFHS group, although they remained higher than in the control group. This decrease suggests the antioxidant activity of Stevia and its ability to mitigate lipid peroxidation, potentially contributing to improved behavioral responses.

Our findings complement those of Simonyan et al. (2021), who demonstrated that Stevia treatment reduced oxidative damage in the nervous tissue of diabetic rats with spinal cord injury. This highlights the potential of Stevia in alleviating lipid peroxidation in the nervous system under various pathological conditions [44].

Additionally, our study aligns with the findings of Hernández García (2018), who reported that Stevia increased glutathione levels and reduced lipid peroxidation in the brains of young rats, despite some observed histological changes. This underscores the need for further investigation into the long-term effects of Stevia on neuronal health, despite its positive impact on oxidative stress [45].

Furthermore, Chavushyan (2017) demonstrated that Stevia reduces NADPH oxidase activity and restores synaptic plasticity in the neurons of rats with fructose-induced metabolic disorders. Our results support these findings, showing that the reduction in oxidative stress in the brain due to Stevia supplementation may contribute to improved neuronal function and behavior [46].

It is important to note that the correlation analysis in our study revealed a strong association between behavioral parameters and lipid peroxidation markers. The increased locomotor activity observed in the HFHS group was closely linked to elevated MDA levels and other markers of lipid peroxidation. The addition of *Stevia rebaudiana* led to a reduction in both behavioral alterations and oxidative stress levels, suggesting a potential mechanism of action through its antioxidant properties.

Studies by Khatun et al. (2021) and Wen et al. (2023) confirm the presence of bioactive compounds in Stevia with potent antioxidant properties, such as luteolin, and its ability to enhance antioxidant enzyme activity while reducing inflammation. These findings support our data, indicating that Stevia may mitigate lipid peroxidation and improve behavioral responses by modulating oxidative stress [47,48].

Furthermore, research by Noreen et al. (2020) and Carrera-Lanestosa et al. (2020) demonstrated that Stevia exhibits antioxidant and antidiabetic effects, reducing MDA levels and increasing superoxide dismutase activity. This highlights its potential in preventing and correcting metabolic disorders, aligning with our observations in rats on an HFHS diet [49,50].

A comparative analysis of the results suggests that *Stachys sieboldii* and *Stevia rebaudiana* exert distinct effects on the organism under a high-fat, high-carbohydrate diet. Stevia exhibits a more pronounced antioxidant effect, reducing biochemical markers of lipid peroxidation (malondialdehyde, conjugated dienes, ketodienes, and Schiff bases) by up to 30–51%. In contrast, Stachys more effectively mitigates hyperactive behavior, as vertical and horizontal activity in the Stachys-treated group decreased by 63–68%, which is significantly greater than in the Stevia-treated group.

Stevia and Stachys contain various bioactive compounds (e.g., phenolic glycosides, flavonoids) that enable them to reduce oxidative stress by directly scavenging free radicals and stimulating cellular antioxidant defense. Experimental studies indicate that Stevia extract significantly increases the activity of key antioxidant enzymes—superoxide dismutase (SOD) and catalase—and elevates reduced glutathione levels, while simultaneously lowering the accumulation of lipid peroxidation products (e.g., TBARS) [51].

In parallel, Stevia modulates inflammatory pathways by decreasing the activity of the transcription factor NF-κB and reducing the production of pro-inflammatory cytokines (TNF-α, IL-1β). This dual effect is mediated via the Nrf2/ARE signaling pathway: Stevia induces Nrf2 expression, thereby enhancing the transcription of antioxidant enzyme genes while concurrently suppressing NF-κB activation [52]. Similarly, Stachys components can initiate intracellular antioxidant responses. For instance, harpagogenin, isolated from *Stachys sieboldii* tubers, has been identified as an activator of the Nrf2/ARE pathway, thereby directly contributing to the antioxidant properties of this plant [53].

As a result, incorporating Stevia and Stachys into the diet boosts the endogenous antioxidant defense system, enabling cells to more effectively neutralize reactive oxygen species (ROS) and protect against oxidative damage to biomolecules.

Beyond reducing free radical levels, these phytocomplexes influence cellular behavior by modulating signaling pathways associated with neuronal survival and function. Lower oxidative stress helps safeguard membrane lipids and proteins from damage, preventing the initiation of apoptotic cascades. Simultaneously, suppression of chronic inflammation (including NF-κB inhibition) alleviates cellular stress responses [52].

These molecular mechanisms manifest in neuronal activity as well. In rats with metabolic disturbances, Stevia prevented pathological alterations in synaptic plasticity: its administration reduced hyperactivation of NADPH oxidase in the hippocampus and amygdala and normalized the potentiation/depression ratio under high-frequency stimulation, illustrating Stevia’s adaptive influence on neural networks under stress [46]. Stachys extracts directly affect neurotransmission: experimental studies show that *Stachys sieboldii* administration enhances GABA receptor activity in hippocampal neurons and increases the expression of neurotrophic factors (NGF, BDNF), which support neuronal survival and synaptic plasticity. This is accompanied by acetylcholinesterase inhibition and strengthened cholinergic transmission, both critical for cognitive functions [54].

Hence, Stevia and Stachys exert neuroprotective effects by jointly impacting antioxidant enzymes, pro-inflammatory transcription factors (Nrf2, NF-κB), and neurotransmitter systems (GABAergic, cholinergic, etc.), ultimately mitigating oxidative stress and stabilizing cellular functions.

Research findings underscore the potential of *Stevia rebaudiana* and *Stachys sieboldii* in preventing and managing diseases linked to oxidative stress. In neurodegenerative conditions—such as Alzheimer’s and Parkinson’s—chronic oxidative stress and neuroinflammation are major contributors to neuronal death [55].

By enhancing antioxidant defenses and inhibiting inflammatory cascades, formulations derived from *Stevia rebaudiana* and *Stachys sieboldii* may slow the progression of neurodegeneration or alleviate its symptoms. For example, in a scopolamine-induced dementia model, Stachys extract helped prevent memory decline, accompanied by elevated brain-derived neurotrophic factor (BDNF) levels and enhanced cholinergic system activity [55]. This effect is similar to that of acetylcholinesterase inhibitors used to treat Alzheimer’s disease, suggesting a potential cognitive benefit of Stachys. Although direct clinical studies on *Stevia rebaudiana* in neurodegenerative diseases remain limited, its neuroprotective properties—demonstrated in metabolic syndrome models (improved synaptic plasticity and decreased NOX activity in the brain)—point to a possible positive effect on patients with insulin resistance or diabetes, both recognized risk factors for Alzheimer’s disease [54].

Regarding Parkinson’s disease, where dopaminergic neuronal loss is largely driven by oxidative stress [56], bolstering antioxidant mechanisms—particularly Nrf2 activation—is considered a promising therapeutic strategy. In this context, *Stevia rebaudiana* and *Stachys sieboldii* attract attention as natural inducers of antioxidant pathways.

In the realm of metabolic disorders, these supplements also yield favorable results. *Stevia rebaudiana* is widely used as a noncaloric sugar substitute, helping to reduce carbohydrate intake and, thus, supporting weight and glycemic control. Beyond replacing sugar, it exhibits direct antidiabetic effects: in diabetic animal models, *Stevia rebaudiana* enhanced glucose tolerance and insulin sensitivity while lowering blood glucose, triglyceride, and cholesterol levels [57].

Moreover, in diabetic mice, *Stevia rebaudiana* supplementation reduced oxidative stress markers (e.g., 4-hydroxynonenal) and the activity of pro-apoptotic pathways, indicating its protective role against diabetes-related complications. Researchers have highlighted *Stevia rebaudiana* as a potential therapeutic agent for diabetic myopathy [58].

Supplements derived from *Stachys sieboldii* also show promise for managing obesity and dyslipidemia. In animals fed a high-calorie diet, *Stachys sieboldii* root powder prevented excessive weight gain and fat accumulation while improving lipid profiles by increasing the excretion of fats and cholesterol [59].

These findings suggest that integrating *Stevia rebaudiana* and *Stachys sieboldii* into the human diet—whether through substituting sugar with *Stevia rebaudiana* or consuming herbal infusions or functional products based on *Stachys sieboldii*—may help counteract the adverse effects of high-calorie diets. Acting on multiple fronts—from improving metabolic parameters (glycemia, lipids) to reducing systemic inflammation and oxidative stress—these natural supplements have the potential to lower the risk of metabolic syndrome and its associated neurocognitive complications. Notably, a clinical trial in healthy volunteers showed that consuming *Stachys lavandulifolia* infusion for only two weeks significantly increased total antioxidant capacity in the blood and reduced lipid peroxidation, underscoring the plant’s preventive potential in conditions linked to oxidative stress [60].

Overall, the results of this study align with previous findings on the neuroprotective effects of plant-based supplements, highlighting several key points. The antioxidant and anti-inflammatory properties of Stevia and other Stachys species are well documented. A meta-analysis of preclinical research demonstrated that *Stevia rebaudiana* leaf extracts substantially restore antioxidant balance (SOD and CAT activity, GSH levels, etc.) in animal models of pathological conditions, thereby reducing oxidative stress markers [51].

Likewise, Stachys has been confirmed to exhibit strong antioxidant capabilities. For instance, a placebo-controlled study found that *Stachys lavandulifolia* infusion significantly increased serum antioxidant capacity and decreased malondialdehyde levels, indicating reduced lipid peroxidation in humans [60].

Similar neuroprotective effects have been noted for other herbal supplements with antioxidant potential. For example, Harada et al. observed that *Stachys sieboldii* extract was as effective as *Ginkgo biloba* extract in preventing ischemic brain damage, reinforcing the idea that boosting endogenous antioxidant defenses is a shared mechanism of neuroprotection among various medicinal plants [27].

Meanwhile, the present study stands out for its comprehensive approach. Previous work has often focused exclusively on the metabolic effects of *Stevia rebaudiana* and related phytotherapeutic agents or separately examined their impact on the nervous system. For example, Han et al. extensively investigated the antidiabetic effects of *Stevia rebaudiana* in mice, demonstrating normalization of glucose and lipids as well as activation of insulin signaling pathways. In contrast, the neuroprotective properties of Stachys were typically studied in models of acute brain injury or cognitive impairment without considering metabolic status [27,57].

Unlike these narrowly focused approaches, our study evaluates the effects of *Stevia rebaudiana* and *Stachys sieboldii* in the context of high-calorie-diet-induced systemic oxidative stress and associated neurobehavioral disorders. This design more clearly establishes the relationship between metabolic improvements (reducing hyperglycemia and lipids) and decreasing neuroinflammation and oxidative damage to neurons. Furthermore, this is the first study to directly compare the effects of *Stevia rebaudiana* and *Stachys sieboldii* within a single experimental framework, whereas previous research typically examined these supplements independently.

The novelty of this approach lies in integrating two promising phytotherapeutic strategies: correcting metabolic dysfunction and providing neuroprotection simultaneously. This offers deeper insights into neuroprotective mechanisms under metabolic stress conditions and underlines the potential value of combining *Stevia rebaudiana* and *Stachys sieboldii* to mitigate the detrimental effects of high-calorie diets.

## 5. Conclusions

The study established that prolonged consumption of a high-fat and high-carbohydrate diet significantly elevates key biochemical markers of oxidative stress in the brain, suggesting enhanced lipid peroxidation that can damage cell membranes and contribute to nervous system dysfunction.

Incorporating bioactive supplements derived from *Stevia rebaudiana* and *Stachys sieboldii* substantially decreased these lipid peroxidation markers, highlighting their strong antioxidant potential. The observed reduction indicates a lessening of oxidative damage in neural tissue and, alongside certain behavioral test outcomes, points to a possible neuroprotective effect of these plant-based components.

The high-fat, high-carbohydrate diet also increased locomotor activity, possibly reflecting heightened excitability or anxiety associated with metabolic disturbances and oxidative stress. Supplementation with Stevia and Stachys partly reduced this excessive movement, whereas grooming and defecation were not significantly affected under either the hypercaloric diet or following plant supplementation.

Correlation analysis revealed robust associations between lipid peroxidation biomarkers and behavioral parameters. In the Stachys group, oxidative stress aligned more closely with autonomic responses, whereas in the Stevia group, a broader interplay emerged, influencing both locomotor patterns and self-regulatory behaviors.

Thus, a high-fat, high-carbohydrate diet not only exacerbates oxidative stress in the brain but also triggers behavioral alterations. Administration of *Stevia rebaudiana* and *Stachys sieboldii* can help counteract these effects, with each plant providing distinct benefits: Stevia more effectively diminishes markers of lipid peroxidation, while Stachys exerts a stronger impact on reducing hyperactive locomotion. This integrated perspective on dietary interventions and plant-based supplementation underscores the potential for targeted or combined use to mitigate adverse outcomes of hypercaloric diets.

## 6. Limitations

Despite the significant findings, this study has several limitations.

The study was conducted on Wistar rats, and while rodent models provide valuable insights into neuroprotective and antioxidant effects, direct extrapolation of the results to human physiology remains uncertain. Differences in metabolism, dietary responses, and cognitive mechanisms between rodents and humans must be considered.

The assessment of locomotor activity in the Open Field Test primarily reflects exploratory behavior rather than direct measures of anxiety or cognitive function. While decreased hyperactivity in rats may suggest behavioral stabilization, it does not necessarily equate to reduced anxiety or improved cognitive function in humans. Further studies incorporating additional behavioral tests (e.g., Elevated Plus Maze, Morris Water Maze) would provide a more comprehensive evaluation.

The study measured lipid peroxidation markers (MDA, conjugated dienes, ketodienes, Schiff bases), but other indicators of oxidative stress, such as protein carbonylation, glutathione levels, and antioxidant enzyme activity (e.g., superoxide dismutase, catalase, glutathione peroxidase), were not assessed. Future studies should incorporate a broader panel of biomarkers to confirm the antioxidant effects of *Stevia rebaudiana* and *Stachys sieboldii*.

Although the results suggest that Stevia and Stachys influence oxidative stress and locomotor behavior, the precise molecular pathways involved remain unclear. Further mechanistic studies, including gene expression and protein activity analysis related to oxidative stress and neurotransmitter regulation, would strengthen the conclusions.

The duration of dietary intervention was limited to 30 days. While this timeframe was sufficient to observe changes in oxidative stress markers and behavior, longer-term studies are needed to evaluate potential cumulative or adaptive effects of chronic supplementation with *Stevia rebaudiana* and *Stachys sieboldii*.

The study used a single dose of Stevia and Stachys, based on recommendations from the literature. However, a dose-dependent analysis would provide more information on the optimal concentrations needed to achieve neuroprotective and antioxidant benefits.

This study focused on biochemical markers and behavioral assessments without conducting histological or neuromolecular analyses of brain tissue. Future research should include immunohistochemistry and Western blot analyses to determine structural and molecular changes in response to supplementation.

## Figures and Tables

**Table 1:** Pearson’s correlation coefficients (r) for the Stachys group.

	Vertical Activity	Horizontal Activity	Grooming	Defecation	MDA	DK	KD	SHO
**Vertical Activity**	1.000	0.925	0.988	0.800	0.692	0.766	0.793	0.859
**Horizontal Activity**	0.925	1.000	0.972	0.968	0.914	0.953	0.965	0.989
**Grooming**	0.988	0.972	1.000	0.882	0.794	0.855	0.877	0.927
**Defecation**	0.800	0.968	0.882	1.000	0.987	0.999	0.999	0.994
**MDA**	0.692	0.914	0.794	0.987	1.000	0.994	0.989	0.964
**DK**	0.766	0.953	0.855	0.999	0.994	1.000	0.999	0.987
**KD**	0.793	0.965	0.877	0.999	0.989	0.999	1.000	0.993
**SHO**	0.859	0.989	0.927	0.994	0.964	0.987	0.993	1.000

**Table 2:** Pearson correlation coefficients (r) for the Stevia group.

	Vertical Activity	Horizontal Activity	Grooming	Defecation	MDA	DK	KD	SHO
**Vertical Activity**	1.000	0.954	0.998	0.996	0.963	0.969	0.999	0.986
**Horizontal Activity**	0.954	1.000	0.970	0.970	0.968	0.999	0.968	0.991
**Grooming**	0.998	0.970	1.000	0.945	0.977	0.981	1.000	0.994
**Defecation**	0.996	0.970	0.945	1.000	0.993	0.990	0.942	0.975
**MDA**	0.963	0.968	0.977	0.993	1.000	0.975	0.980	0.994
**DK**	0.969	0.999	0.981	0.990	0.975	1.000	0.980	0.997
**KD**	0.999	0.968	1.000	0.942	0.980	0.980	1.000	0.993
**SHO**	0.986	0.991	0.994	0.975	0.994	0.997	0.993	1.000

## Data Availability

All data generated or analyzed during this study are included in this published article.

## Abbreviations

The following abbreviations are used in this manuscript:

HFHS	High-fat, high-sugar
MDA	Malondialdehyde
DK	Conjugated dienes
KD	Ketodienes
SHO	Schiff bases
FDA	U.S. Food and Drug Administration
ANOVA	Analysis of variance
TBA	Thiobarbituric acid
UV–Vis	Ultraviolet–visible (spectroscopy)
rpm	Revolutions per minute
*w*/*v*	Weight/volume ratio
*v*/*v*	Volume/volume ratio
pH	Hydrogen ion concentration
r	Pearson’s rank correlation coefficient
RU/ml	Relative units per milliliter
μmol/ml	Micromoles per milliliter
